# Anthropometric and Physical Qualities of Elite Male Youth Rugby League Players

**DOI:** 10.1007/s40279-017-0745-8

**Published:** 2017-06-03

**Authors:** Kevin Till, Sean Scantlebury, Ben Jones

**Affiliations:** 1 Institute for Sport, Physical Activity and Leisure, Leeds Beckett University, Headingley Campus, Room 108, Cavendish Hall, West Yorkshire, Leeds, LS6 3QS UK; 2 Leeds Rhinos RLFC, Leeds, UK; 3 Rugby Football League, Red Hall, Leeds, UK

## Abstract

Rugby league is a collision team sport played at junior and senior levels worldwide, whereby players require highly developed anthropometric and physical qualities (i.e. speed, change-of-direction speed, aerobic capacity, muscular strength and power). Within junior levels, professional clubs and national governing bodies implement talent identification and development programmes to support the development of youth (i.e. 13–20 years) rugby league players into professional athletes. This review presents and critically appraises the anthropometric and physical qualities of elite male youth rugby league players aged between 13 and 20 years, by age category, playing standard and playing position. Height, body mass, body composition, linear speed, change-of-direction speed, aerobic capacity, muscular strength and power characteristics are presented and demonstrate that qualities develop with age and differentiate between playing standard and playing position. This highlights the importance of anthropometric and physical qualities for the identification and development of youth rugby league players. However, factors such as maturity status, variability in development, longitudinal monitoring and career attainment should be considered to help understand, identify and develop the physical qualities of youth players. Further extensive research is required into the anthropometric and physical qualities of youth rugby league players, specifically considering national standardised testing batteries, links between physical qualities and match performance, together with intervention studies, to inform the physical development of youth rugby league players for talent identification and development purposes.

## Key Points

**Table:** 

Anthropometric and physical qualities develop with age in junior rugby league players but this is influenced by maturity status and the individual variability in the development of such qualities.
Anthropometric and physical qualities differ between playing standard, with increased physical qualities related to higher playing levels and future career success.
Anthropometric and physical qualities differ between playing positions, with forwards generally bigger with greater strength, while backs have greater linear speed, change-of-direction speed and aerobic capacity.

## Introduction

Rugby league is a collision team sport played at junior and senior levels worldwide. Professional teams and game popularity are most established in the UK, France, Australia and New Zealand [1, 2], with the European Super League and Australasian National Rugby League (NRL) the two major professional leagues in the world. Rugby league teams consist of 13 players commonly split into two major playing groups (i.e. ‘backs’ and ‘forwards’) or four subgroups of outside backs (fullback, wing, centre), pivots (stand-off, scrum-half, hooker), props, and back row (second row, loose forward). The playing demands vary by position, with outside backs involved in more free running, pivots undertaking greater decision making and ball handling roles, and props and back-row positions involved in more physical collisions [2, 3].

Rugby league performance may be determined by the complex interaction of individual players’ technical, tactical, cognitive and physical qualities [4]. To date, the majority of available research has focused on the physical demands of match-play and the physical qualities of players, which has recently been comprehensively summarised in a review titled ‘Applied Sport Science of Rugby League’ [5]. Research shows rugby league match-play is intermittent, involving frequent periods of high-intensity activity (e.g. high-speed running) separated by lower-intensity activity (e.g. jogging, repositioning) [6–8]. Total distance covered during match-play can range between 4000 and 8000 m dependent on playing position and playing standard [6, 9, 10], with up to 1000 m covered at high-speed distances [8] comprising a large number of short distance (i.e. 10 m) efforts [11]. As well as the large high-speed running demands, players are frequently involved in a large number of collision and wrestling bouts through defensive (e.g. tackling) and offensive (ball carrying) involvements [12]. Due to the high physical demands of rugby league, players require highly developed anthropometric and physical qualities (i.e. linear speed, change-of-direction speed, aerobic capacity, muscular strength and power) to succeed [3, 5].

Although Johnston and colleagues [5] reviewed rugby league research, only a brief summary was provided for the youth rugby league player. However, the identification and development of the next generation of youth rugby league players is a major focus of rugby league national governing bodies and professional clubs. Thus, talent identification and development programmes are common practice, usually termed rugby league academies [13, 14], to support the development and transition of youth rugby league players aged between 13 and 20 years into senior professional athletes. The physical development of youth rugby league players is of vast importance to player development staff, coaches, sport scientists, and strength and conditioning practitioners in advancing players to meet the high physical demands associated with professional rugby league match-play [5]. Therefore, the use of objective markers of physical development and evidence-based practices to support talent identification and development is a very important consideration in the optimisation of the development of youth rugby league players, as it is with other sports (i.e. taekwondo [15] and mixed martial arts [16]). In addition, understanding factors that may influence the development of physical characteristics (i.e. growth, maturation) during this key development period (i.e. adolescence) is important for optimising long-term development within youth rugby league players. Therefore, the collation of existing research to provide a clear understanding of the importance and development of physical qualities for youth rugby league players would be beneficial for research and practice.

Therefore, the purpose of this review was to (1) present the anthropometric and physical qualities of elite male youth rugby league players aged between 13 and 20 years; and (2) critically appraise the literature surrounding the anthropometric and physical qualities of youth rugby league players, drawing comparisons between age categories, playing standards and playing positions while considering factors such as growth, maturation and longitudinal development. This review provides a framework to assist practitioners to effectively prepare youth players for the physiological demands of professional rugby league while understanding factors that may affect the physical development of the youth rugby league player.

## Methodological Aspects

A computer literature search of the PubMed, Google Scholar, and Scopus electronic databases was performed for English-language, peer-reviewed articles from inception to April 2016 using the following key words: ‘rugby league’, ‘youth AND rugby league’, ‘elite AND youth AND rugby league’, ‘anthropometric AND rugby league’, ‘body composition AND rugby league’, ‘speed AND rugby league’, ‘agility AND rugby league’, ‘change of direction speed AND rugby league’, ‘power AND rugby league’, ‘aerobic capacity AND rugby league’, and ‘strength AND rugby league’. The electronic search was supplemented by hand searching the reference lists for articles that met the study inclusion criteria.

The themes of the review represented the major anthropometric and physical qualities influencing rugby league performance, including height, body mass, body composition, linear speed, change-of-direction speed (agility), aerobic capacity, muscular strength and muscular power. Only those studies examining the abovementioned fitness physical qualities using established and accepted methods in the context of athlete preparation and performance were included. Data are presented in tables and discussed in the text. As the review sought to identify physical qualities within elite youth rugby league players, studies that only investigated sub-elite junior rugby league players were excluded from the data tables but were included in the review text. Elite players were defined as players who were selected for a national governing body talent identification and development programme or were members of a professional rugby league club academy programme. Studies that reported data on elite players within annual-age categories for all players (regardless of position) or by positional groups (i.e. forwards vs. backs) or subcategories (e.g. outside backs, pivots, props, back row) are presented in the tables. After the exclusion of articles that did not meet the required criteria, the tables present the anthropometric characteristics from 12 articles; linear speed, change-of-direction speed, aerobic capacity and muscular power from 11 articles, while strength characteristics were reviewed from 4 articles. Other articles are discussed throughout the text but are not included in the presentation of data in the tables.

## Anthropometric Qualities

Due to the physical contact nature of rugby league, anthropometric qualities are deemed important for performance [17, 18]. Table 1 presents the height and body mass data for elite youth rugby league players by age category (under 13–20s) and playing position.

**Table 1:** Anthropometric qualities of elite youth rugby league players categorised by age and playing position

Age category (years)	Position	Height (cm)	Body mass (kg)	Sum of skinfolds (mm)
Under 13	All–regional [13]^a^	169.6 ± 8.4	62.4 ± 11.4	38.6 ± 16.4^b^
	All–national [13]^a^	171.0 ± 7.1	63.7 ± 9.0	34.9 ± 12.3^b^
	All [20]	171.2 ± 7.0	63.9 ± 9.8	36.2 ± 15.0^b^
	Backs [17]			
	Outside backs	171.5 ± 6.8	60.3 ± 6.1	26.9 ± 5.8^b^
	Pivot	165.0 ± 5.6	55.5 ± 7.1	31.9 ± 11.0^b^
	Forwards [17]			
	Props	174.0 ± 7.5	75.0 ± 8.8	52.3 ± 18.6^b^
	Back row	174.2 ± 4.6	67.2 ± 6.7	39.0 ± 12.1^b^
Under 14	All–regional [13]^a^	175.0 ± 6.5	70.2 ± 10.8	40.1 ± 17.1^b^
	All–national [13]^a^	175.3 ± 6.5	71.1 ± 9.3	35.8 ± 12.2^b^
	All [20]	175.7 ± 6.2	71.1 ± 9.6	39.3 ± 15.0^b^
	Backs [17]			
	Outside backs	175.7 ± 6.4	67.9 ± 5.3	32.2 ± 10.7^b^
	Pivot	171.1 ± 4.6	63.2 ± 7.9	34.4 ± 13.9^b^
	Forwards [17]			
	Props	178.1 ± 6.9	81.5 ± 8.6	53.2 ± 13.6^b^
	Back row	178.3 ± 4.0	74.3 ± 7.4	41.5 ± 14.3^b^
Under 15	All [26]	173.0 ± 3.0	73.9 ± 5.5	
	All–regional [13]^a^	177.8 ± 6.3	75.8 ± 10.9	41.1 ± 15.8^b^
	All–national [13]^a^	178.3 ± 6.4	77.6 ± 9.7	39.9 ± 14.5^b^
	All [19]	169.5 ± 2.1	69.4 ± 2.5	72.2 ± 8.7^c^
	All [20]	178.6 ± 5.7	77.6 ± 9.8	42.4 ± 16.0^b^
	All [23]		81.9 ± 9.1	83.9 ± 30.3^d^
	Backs [17]			
	Outside backs	178.3 ± 6.4	73.4 ± 6.3	33.9 ± 8.9^b^
	Pivot	175.0 ± 4.1	70.0 ± 6.3	35.6 ± 12.5^b^
	Forwards [17]			
	Props	180.2 ± 6.6	88.5 ± 9.3	58.8 ± 16.5^b^
	Back row	180.7 ± 4.0	80.7 ± 7.3	45.3 ± 14.9^b^
Under 16	All [23]		86.1 ± 6.0	81.0 ± 25.0^d^
	All [34]	177.4 ± 2.7	77.9 ± 9.8	37.9 ± 12.9^b^
	Backs [21]	173.1 ± 8.2	68.4 ± 8.6	30.4 ± 5.9^b^
	Forwards[21]	177.7 ± 5.4	80.9 ± 9.7	42.7 ± 14.1^b^
	Backs [25]	178.8 ± 5.5	74.9 ± 7.6	
	Forwards [25]	180.9 ± 6.7	87.0 ± 11.1	
	All [26]	177.0 ± 2.0	82.1 ± 4.2	
	All [59]	178.0 ± 4.4	83.2 ± 9.8	
Under 17	All [23]		86.3 ± 9.4	77.8 ± 20.8^d^
	All [34]	178.8 ± 2.9	84.8 ± 10.1	41.4 ± 14.0^b^
	Backs [21]	173.4 ± 4.1	75.4 ± 7.0	31.4 ± 6.2^b^
	Forwards [21]	180.5 ± 5.5	85.7 ± 8.6	40.5 ± 15.1^b^
	All [26]	177.0 ± 4.0	80.1 ± 5.5	
Under 18	All [19]	179.7 ± 1.3	77.9 ± 1.9	73.8 ± 4.1^c^
	All [34]	179.7 ± 2.7	86.6 ± 9.1	38.3 ± 11.2^b^
	All [59]	182.0 ± 5.3	85.3 ± 9.6	
	Backs [21]	176.7 ± 5.9	78.5 ± 7.6	32.2 ± 7.9^b^
	Forwards [21]	181.9 ± 4.6	90.9 ± 8.1	42.9 ± 12.8^b^
	Backs [32]	178.6 ± 5.5	80.9 ± 7.1	
	Forwards [32]	180.6 ± 6.6	92.6 ± 12.2	
Under 19	All [34]	180.2 ± 2.7	88.0 ± 9.4	36.4 ± 10.4^b^
	Backs [21]	179.3 ± 5.4	81.8 ± 8.0	30.5 ± 6.9^b^
	Forwards [21]	182.7 ± 5.0	94.1 ± 7.7	43.5 ± 14.2^b^
Under 20	Backs [21]	176.8 ± 6.1	82.8 ± 6.3	
	Backs [21]	180.1 ± 4.9	85 ± 6.3	31.9 ± 7.4^b^
	Forwards [21]	180.1 ± 7.7	90.1 ± 11.7	
	Forwards [21]	180.1 ± 5.7	92.6 ± 8.8	39.5 ± 9.1^b^

Data are expressed as mean ± standard deviation

^a^Both regional and national levels were included because these were defined as elite in this study

^b^Sum of four skinfolds

^c^Sum of seven skinfolds

^d^Sum of six skinfolds

Height and body mass have been shown to increase with age in elite [17, 19–23] and sub-elite players [24, 25], are greater within elite compared with sub-elite levels [13, 26–28], and are higher in forwards than backs [17, 21, 29, 30]. Findings in elite players aged between 13 and 15 years [17] showed that 92.4 and 33.3% of players were taller and 96.0 and 30.3% were heavier than the UK 50th and 97th growth percentiles, respectively [31]. Similar findings, although less striking, were apparent in Australian under 18 players, with 24 and 4% above the 90th and 97th percentiles for height, respectively, and 25% of players above the 97th percentile for body mass [32]. Therefore, youth players are taller and heavier than the general population, with greater size an advantage for participation and performance in youth rugby league.

Height and body mass increase with age due to the normal processes related to growth and maturation [33]. Longitudinal data in players aged under 13–15 [20] and under 16–20 [34] have shown annual increases in height and body mass into the early 20s, with greater gains occurring at younger ages (i.e. 13–15 years) [3, 20, 34, 35] due to maturational processes (see Sect. 10). Interestingly, currently available research (Table 1) within elite youth players shows shorter and lighter players in the under 16 compared with under 15 age categories. This occurs due to the under 15s being a national talent development programme, compared with the under 16s being from an elite club academy, and is a limitation of the current research.

When comparing height and body mass between playing standards, increased size provided a small advantage for selection between elite (178.0 ± 5.9 cm, 77.5 ± 10.0 kg) and sub-elite (175.2 ± 6.9 cm, 72.3 ± 11.7 kg) levels in players in the under 16 age category [28], and a small to moderate difference between starters and non-starters in both elite and sub-elite levels at under 14, 16 (e.g. body mass 77.5 ± 10.0 vs. 74.3 ± 13.4 kg) and 18 age categories [25]. Senior professional players from two European Super League clubs were also taller (183.2 ± 5.8 vs. 179.2 ± 5.7 cm) and heavier (96.5 ± 9.3 vs. 86.5 ± 9.0 kg) than their academy under 19 counterparts [30]. Such findings suggest that increased size is advantageous for selection to higher playing standards in rugby league, most probably due to the ability to generate greater impact forces throughout the frequent contacts involved in the sport [5, 18]. However, within more homogenous samples (i.e. regional and national level within the UK between under 13s and 15s), no differences were observed for height and body mass between playing standards (see Table 1) [13]. In addition, when comparing height and body mass of 13- to 15-year-olds against future career attainment (i.e. whether players achieved amateur, academy or professional status in adulthood), no differences were found between career attainment levels [14, 36], and, in the under 14 age category, future professionals (61.7 ± 9.2 kg) had a significantly lower body mass than amateur (71.1 ± 11.8 kg) and academy (70.0 ± 10.7 kg) players [36]. These differences may be supported by the fact that no relationships were observed between height and body mass and tackling ability in junior players despite contrasting findings in senior cohorts [37]. Contradictory to these findings, within players in the under 17–19 age categories from a professional UK Super League club, height (e.g. under 17s, 176.9 ± 5.5 vs. 181.8 ± 3.1 cm) and body mass (e.g. under 19s, 87.5 ± 9.9 vs. 90.8 ± 9.7 kg) did differentiate between players who achieved professional or academy status [38]. Therefore, increased body size may not be advantageous until post 16 years of age, when increased height and development of body mass may influence player development and future career success because of the demands of the sport.

Positional differences in body size are consistent with findings in senior players, with forwards (i.e. props and back row, or hit-up forwards) being taller and heavier than backs (i.e. pivots and outside backs) [17, 21, 30, 39]. These findings are aligned to the match demands of forwards and backs in senior competition [6], with increased frequency of collisions within forward positions alongside the importance of winning the ruck. However, limited match characteristics are available by playing position in youth players and little is known about the impact that this may have on player development.

In summary, height and body mass are deemed important for rugby league performance, increasing with age and generally differentiating between playing standard and position. However, body size may not be as important a quality in younger players (under 16 years) due to variations in maturity. It is recommended that practitioners are aware of the variability in the development of height and body mass into adulthood, and track such measures in their players into the early 20s. In addition, data on further anthropometric characteristics such as limb length, limb circumference and muscle cross-sectional area [23], as well as somatotype [32], are limited in current research and may provide a more detailed evaluation of the anthropometric characteristics important for player development within youth rugby league players. Other factors such as ethnicity also require further consideration [32].

## Body Composition

Body composition is another important consideration for physical performance in rugby league and has primarily been assessed via the use of sum of skinfold assessments (see Table 1). Sum of skinfolds generally does not differentiate between age categories [17, 19–21, 23, 25] but does differ between junior and senior sub-elite players [39], is reduced in elite compared with sub-elite players [13, 27, 28], and is greater in forwards than backs [17, 21, 32, 39]. Research findings generally show sum of four skinfold values of 30 and 40 mm in backs and forwards, respectively [21], with recent research using dual X-ray absorptiometry (DXA) demonstrating body composition values of 16 and 20% for backs and forwards, respectively [30].

The lack of differences between age categories [17, 19–21, 23, 25] and limited change in longitudinal data in players in the under 13–15 [20, 22] and under 16–20 age categories [34, 38] suggest greater stability of skinfolds with age than with height and body mass. However, studies examining seasonal changes in sum of skinfolds [35, 40] show reductions during the season. Therefore, body fat percentage may be a more variable anthropometric quality that can regularly change related to an individual’s training status and nutritional intake and should therefore be monitored regularly.

Comparing body composition at different playing standards showed that a reduced sum of skinfolds provides a small advantage for elite versus sub-elite players (e.g. 67.1 ± 14.8 vs. 76.4 ± 28.1 mm) [27, 28] and regional versus national-level players (see Table 1) [13]. In comparisons between academy and professional levels, after controlling for height and body mass, backs showed no differences in fat mass (e.g. 13.7 ± 1.6 vs. 12.6 ± 1.1 kg), whereas large differences were found in total fat (19.3 ± 1.6 vs. 15.4 ± 1.1 kg), lean mass, and bone mineral content (BMC), whereby these differences were particularly favourable in the legs of professional-level forwards [30]. When considering future career attainment level, future professionals (33.4 ± 9.8 mm) had reduced skinfolds compared with amateur players (41.6 ± 18.2 mm) in the under 13–15 age categories [14, 36]. However, reduced skinfolds did not differentiate between future professional and academy players in the under 17–19 age categories (e.g. under 19s, 38.4 ± 15.6 vs. 36.9 ± 8.5 mm) [38]. Generally, such findings for body composition suggest that reduced body fat percentage seems advantageous for selection to higher playing standards in youth rugby league players. This is consistent with senior players in whom skinfold thickness has been shown to predict selection [41] and be related to the frequency of completed and dominant tackles during match-play [42]. Explanations for this finding could be due to physiological mechanisms (e.g. power-to-mass ratio, thermoregulation) [2, 3], relationships to physical measures (e.g. speed, agility) [17], or increased training or playing status at higher levels, but further evidence is required to substantiate this.

Comparisons between positions suggest forwards tend to carry more body fat than backs [17, 21, 30, 32, 39], with props usually having greater body fat than back-row positions [17]. Consistent with height and body mass, these findings are aligned with match demands in senior competition [24], including reduced playing minutes but greater collisions in forwards than backs. It has been suggested that increased fat may be a protector against injury [24], but no evidence is available to support this.

In summary, appropriate body fat percentage is important for rugby league performance, differentiates between playing standard and position, and should be a consideration for player development of youth rugby league players. However, optimum skinfold/body fat percentage scores are unknown and it may be appropriate to control body fat levels, with four site skinfolds recommended at approximately 30 mm in backs and 40 mm in forwards [21]. Practitioners should be aware of large individual variability in body fat percentage and should monitor body fat levels regularly in youth players, using internationally standardised methods (i.e. International Society for Assessment of Kinanthropometry [ISAK] [32] or DXA [30]), which are lacking within the existing evidence base.

## Linear Speed

The ability to move fast in attack and defence is an important aspect of rugby league performance. Linear speed data for elite youth rugby league players over 10, 20, 30, 40 and 60 m are presented in Table 2. Linear speed has been shown to differentiate between age categories within some studies [17, 20, 23–26] but not others [19, 21]. Differences between playing level [13, 24–28] and playing position [17, 21, 24, 26, 39, 43] have consistently been identified, highlighting greater speed in elite compared to sub-elite players, and backs compared to forwards.

**Table 2:** Linear speed, change-of-direction speed, muscular power and estimated $\dot{V}{\text{O}}_{2\hbox{max} }$ qualities off elite youth rugby league players categorised by age and playing position

Age category (years)	Position	10 m (s)	20 m (s)	30 m (s)	40 m (s)	60 m (s)	Agility 5-0-5, left (s)	Agility 5-0-5, right (s)	CMJ (cm)	MBT (m)	Aerobic capacity
Under 13	All–regional [13]^a^	1.95 ± 0.05	3.38 ± 0.20	4.75 ± 0.26		8.89 ± 0.56	2.59 ± 0.14	2.61 ± 0.14	38.2 ± 5.1	5.1 ± 0.7	46.4 ± 4.7^b^
	All–national [13]^a^	1.91 ± 0.12	3.29 ± 0.14	4.61 ± 0.22		8.59 ± 0.47	2.55 ± 0.13	2.57 ± 0.14	39.6 ± 5.0	5.4 ± 0.6	49.5 ± 3.7^b^
	All [20]	1.96 ± 0.08	3.36 ± 0.16	4.70 ± 0.23		8.76 ± 0.54	2.56 ± 0.13	2.57 ± 0.15	38.9 ± 5.0	5.4 ± 0.6	47.9 ± 5.4^b^
	Backs [17]										
	Outside backs	1.92 ± 0.09	3.28 ± 0.15	4.57 ± 0.23		8.44 ± 0.46	2.55 ± 0.12	2.55 ± 0.15	42.2 ± 4.8	5.2 ± 0.6	50.8 ± 3.8^b^
	Pivots	1.95 ± 0.09	3.38 ± 0.16	4.74 ± 0.24		8.88 ± 0.56	2.57 ± 0.17	2.56 ± 0.16	38.4 ± 4.5	4.9 ± 0.7	49.1 ± 3.7^b^
	Forwards [17]										
	Props	2.01 ± 0.08	3.47 ± 0.16	4.85 ± 0.24		9.10 ± 0.58	2.61 ± 0.12	2.62 ± 0.12	35.9 ± 4.1	5.4 ± 0.6	42.4 ± 7.2^b^
	Back row	1.95 ± 0.06	3.34 ± 0.11	4.72 ± 0.15		8.82 ± 0.39	2.55 ± 0.12	2.58 ± 0.13	37.2 ± 4.0	5.5 ± 0.6	47.4 ± 3.4^b^
Under 14	All–regional [13]^a^	1.91 ± 0.09	3.29 ± 0.25	4.58 ± 0.22		8.49 ± 0.46	2.49 ± 0.14	2.51 ± 0.15	40.3 ± 5.3	5.8 ± 0.7	48.7 ± 5.3^b^
	All–national [13]^a^	1.87 ± 0.10	3.21 ± 0.15	4.50 ± 0.21		8.32 ± 0.44	2.44 ± 0.12	2.46 ± 0.11	41.9 ± 5.1	6.0 ± 0.5	50.9 ± 3.9^b^
	All [20]	1.91 ± 0.08	3.26 ± 0.14	4.53 ± 0.20		8.39 ± 0.41	2.47 ± 0.10	2.48 ± 0.11	41.3 ± 4.4	5.9 ± 0.5	50.1 ± 4.7^b^
	Backs [17]										
	Outside backs	1.87 ± 0.07	3.18 ± 0.11	4.41 ± 0.15		8.14 ± 0.30	2.43 ± 0.10	2.44 ± 0.11	43.8 ± 3.7	5.8 ± 0.6	51.8 ± 4.6^b^
	Pivots	1.92 ± 0.10	3.28 ± 0.17	4.56 ± 0.25		8.49 ± 0.52	2.47 ± 0.07	2.47 ± 0.11	40.0 ± 5.4	5.5 ± 0.6	50.1 ± 3.8^b^
	Forwards [17]										
	Props	1.95 ± 0.07	3.34 ± 0.11	4.66 ± 0.17		8.65 ± 0.35	2.59 ± 0.08	2.6 ± 0.08	39.1 ± 4.0	6.1 ± 0.6	46.2 ± 4.3^b^
	Back row	1.90 ± 0.07	3.26 ± 0.12	4.54 ± 0.17		8.39 ± 0.34	2.46 ± 0.10	2.47 ± 0.10	41.1 ± 3.4	6.0 ± 0.6	50.8 ± 4.0^b^
Under 15	All–regional [13]^a^	1.86 ± 0.14	3.19 ± 0.17	4.43 ± 0.22		8.17 ± 0.41	2.46 ± 0.14	2.48 ± 0.13	42.5 ± 5.4	6.4 ± 0.7	50.6 ± 4.8^b^
	All–national [13]^a^	1.85 ± 0.14	3.17 ± 0.17	4.40 ± 0.23		8.10 ± 0.43	2.45 ± 0.16	2.47 ± 0.14	43.5 ± 5.0	6.5 ± 0.6	51.1 ± 3.6^b^
	All [19]^c^	1.84 ± 0.02	3.17 ± 0.03		5.69 ± 0.06		2.48 ± 0.03^d^		42.0 ± 2.0		48.8 ± 1.1^b^
	All [20]	1.87 ± 0.08	3.18 ± 0.12	4.41 ± 0.19		8.10 ± 0.41	2.44 ± 0.13	2.47 ± 0.14	43.4 ± 5.1	6.5 ± 0.6	51.3 ± 4.5^b^
	All [23]		3.50 ± 0.10						47.0 ± 3.0		48.1 ± 3.4^b^
	Backs [17]										
	Outside backs	1.83 ± 0.07	3.10 ± 0.11	4.28 ± 0.16		7.80 ± 0.35	2.40 ± 0.11	2.41 ± 0.11	46.1 ± 4.3	6.5 ± 0.7	51.8 ± 4.6^b^
	Pivots	1.87 ± 0.07	3.19 0.10	4.45 ± 0.17		8.19 ± 0.35	2.45 ± 0.12	2.46 ± 0.14	42.3 ± 5.7	6.1 ± 0.6	52.3 ± 3.4^b^
	Forwards [17]										
	Props	1.91 ± 0.07	3.25 ± 0.11	4.48 ± 0.17		8.30 ± 0.29	2.49 ± 0.17	2.55 ± 0.17	40.3 ± 4.0	6.7 ± 0.6	48.0 ± 5.0^b^
	Back row	1.88 ± 0.08	3.21 ± 0.11	4.49 ± 0.17		8.27 ± 0.39	2.42 ± 0.13	2.47 ± 0.14	43.7 ± 4.8	6.6 ± 0.6	52.6 ± 4.1^b^
Under 16	All [26]	1.89 ± 0.04			5.65 ± 0.10		2.45 ± 0.04^d^		50.0 ± 1.9		10.6 ± 0.5^e^
	All [23]		3.40 ± 0.20						47.3 ± 4.9		48.3 ± 3.6^b^
	All [34]	1.82 ± 0.07	3.12 ± 0.12						44.1 ± 3.8		1286 ± 493^f^
	All [26]	1.81 ± 0.03			5.42 ± 0.11		2.40 ± 0.05^d^		53.1 ± 3.6		11.3 ± 0.7^e^
	Backs [17]	1.78 ± 0.06	3.07 ± 0.11						48.1 ± 4.6		47.5 ± 3.0^g^
	Forwards [17]	1.85 ± 0.06	3.18 ± 0.09						43.8 ± 5.0		47.1 ± 3.7^g^
	Backs [25]	1.76 ± 0.09	3.03 ± 0.12		5.40 ± 0.22		2.27 ± 0.12^d^		47.5 ± 4.6		54.6 ± 8.5^b^
	Forwards [25]	1.85 ± 0.06	3.18 ± 0.09		5.69 ± 0.17		2.34 ± 0.15^d^		48.9 ± 4.1		48.6 ± 5.1^b^
Under 17	All [23]		3.30 ± 0.10						47.6 ± 5.5		52.2 ± 3.5^b^
	All [34]	1.81 ± 0.05	3.11 ± 0.09						47.8 ± 5.6		1308 ± 347^f^
	All [26]	1.83 ± 0.05			5.46 ± 0.14		2.36 ± 0.08^d^		58.9 ± 3.8		12.3 ± 0.8^e^
	Backs [21]	1.78 ± 0.04	3.07 ± 0.07						50.5 ± 6.0		48.5 ± 2.1^g^
	Forwards [21]	1.83 ± 0.07	3.16 ± 0.11						48.0 ± 5.6		48.9 ± 3.2^g^
Under 18	All [19]^c^	1.87 ± 0.02	3.24 ± 0.04		5.83 ± 0.08		2.44 ± 0.02^d^		42.8 ± 1.1		45.2 ± 1.2^b^
	All [34]	1.81 ± 0.05	3.10 ± 0.11						51.3 ± 6.0		1502 ± 301^f^
	Backs [21]	1.78 ± 0.05	3.06 ± 0.09						52.6 ± 5.7		49.1 ± 2.2^g^
	Forwards [21]	1.81 ± 0.06	3.11 ± 0.11						49.1 ± 5.2		48.8 ± 3.3^g^
Under 19	All [34]	1.80 ± 0.05	3.08 ± 0.09						52.1 ± 5.3		1667 ± 406^f^
	Backs [21]	1.79 ± 0.07	3.04 ± 0.08						54.0 ± 6.2		48.9 ± 2.7^g^
	Forwards [21]	1.83 ± 0.08	3.14 ± 0.12						51.2 ± 4.4		48.3 ± 3.2^g^
Under 20	Backs [21]	1.99 ± 0.06	3.26 ± 0.07		5.55 ± 0.13				50.6 ± 5.0		
	Forwards [21]	2.06 ± 0.10	3.39 ± 0.17		5.80 ± 0.26				50.6 ± 7.1		
	Backs [21]	1.76 ± 0.07	2.99 ± 0.10						54.4 ± 6.1		49.1 ± 2.8^g^
	Forwards [21]	1.82 ± 0.04	3.16 ± 0.07						51.0 ± 4.1		48.6 ± 3.0^g^

Data are expressed as mean ± standard deviation

*VO*_*2max*_ maximum oxygen uptake, *CMJ* countermovement jump, *MBT* medicine ball chest throw

^a^Both regional and national levels were included because these were defined as elite in this study

^b^Estimated VO_2max_ (ml kg^−1^ min^−1^) from multistage fitness test

^c^Post-training data are presented for this study

^d^These studies provide only one agility 5-0-5 score therefore direction is not available

^e^Multistage fitness test level

^f^Yo-Yo test distance (m)

^g^Estimated VO_2max_ (ml kg^−1^ min^−1^) from Yo-Yo test

Current research suggests that linear speed can differentiate between age categories within the younger age categories (i.e. under 16 years) [17, 23, 24, 26] but is less likely to differentiate between older age groups (i.e. over 16 years) [21]. Studies assessing linear speed development longitudinally in younger players [20, 22] demonstrate improvements between the under 13 and 15 age categories. This is likely due to the normal adaptations related to growth and maturation [33], with the changes in height during this period that influence stride length and rate improving linear speed. Furthermore, longitudinal speed development at the 60 m distance according to earlier or later maturing players demonstrated greater improvement in later maturing players aged 13–15 years [22]. Studies in older youth players (i.e. over 16 years of age) showed no significant changes in speed across 10- [19] and 14-week [44] training periods, across a season [35, 40], annually, and long term (i.e. 4 years) [34]. This may occur due to the process of peak weight velocity, which follows peak height velocity (PHV), whereby heightened gains in body mass occur. Gains in body mass and reductions in the changes in height may reduce speed development, suggesting that the assessment of momentum (i.e. body mass × velocity), alongside speed, may be an important consideration for monitoring and evaluation of players, especially post 16 years of age. Momentum has been shown to differentiate between age categories [21] and improve with age [34], which may be due to increases in mass and maintenance of speed in older youths, but to a combination of improvements in both sprint speed and mass in younger players.

Linear speed has differentiated between elite and sub-elite players [25–28] (e.g. 40 m; under 17, 5.92 ± 0.14 vs. 5.46 ± 0.14 s) [26], national and regional players (see Table 2) [13], starters and non-starters [25] and future career attainment levels (e.g. 20 m; professionals 3.21 ± 0.16 vs. amateurs 3.29 ± 0.19 s) [14]. This is consistent with findings in senior players [24], and suggests that advanced speed is an important physical quality for higher playing standard and career attainment. Advanced acceleration has been associated with enhanced tackling ability in 16-year-old elite and sub-elite players [28], suggesting acceleration contributes to successful tackle performance and proficiency. In addition, the development of momentum (professionals 47 vs. academy 17 kg s^−1^) between under 17s and 19s [38] contributed to attaining professional status. Momentum may be linked to acceleration and has been shown to be related to successful carries per minute in under 15s, 16s and 17s [45]. This is suggested to advance ball carrying at the gain line, allow greater force in overcoming opposing defenders, greater impact forces in tackles and potential increases in speed of playing the ball.

Backs have been shown to be quicker than forwards across a range of distances [21, 24, 26, 39, 43]. When considered in positional subgroups, pivots are quicker over 10 m, while outside backs are quicker over longer distances. Props are generally slower than all positions [17]. Although forwards have reduced speed compared with backs, this may be due to their greater mass, resulting in increased momentum [21]. Such findings are related to the positional demands, with forwards involved in more collision-based activities for which momentum may be more important. Backs may engage in greater free running for which speed over greater distances may be more important.

In summary, linear speed is important for rugby league performance and differentiates between age categories at younger ages (i.e., under 16 years), playing standard and playing position. However, speed does not seem to increase with age within older (i.e. over 16 years) age categories. In addition, momentum, especially over shorter distances, is a physical quality related to rugby league performance that differentiates between age, standard and position. Therefore, both linear speed and momentum are key aspects for physical development within youth rugby league players aged between 13 and 20 years, and should be evaluated, monitored and developed. However, research to date is limited in relation to the optimum training programmes for developing speed and should therefore be a consideration of future research studies.

## Change-of-Direction Speed

The movement patterns of rugby league involve a combination of accelerations, decelerations and changes in direction [46]. Therefore, the ability to change direction is important. Change-of-direction speed has been assessed via a range of measures, including the ‘L’ run [39], Illinois agility run [24] and the agility 5-0-5 test [17, 47]. Based on the inclusion criteria for the study, change-of-direction speed data are presented in Table 2 for the 5-0-5 test only. Change-of-direction speed has been shown to differentiate between age categories for Illinois agility [24] and agility 5-0-5 performance [17, 20, 25, 26] but not in all studies [19]. Differences between playing level [13, 25–28] demonstrate elite players outperform sub-elite players, but differences between backs and forwards are generally limited [24, 26, 39], except for props being the worst-performing position [17].

Like other physical qualities, change-of-direction speed has generally increased with age [17, 24–26], with longitudinal studies demonstrating an improvement in change-of-direction speed between 13 and 15 years of age [20]. Furthermore, improvements in players aged 17–19 years demonstrated approximately 6% improvements following a 14-week preseason training programme including two field sessions per week [44] and over a competitive season [48]. Differences between age categories and improvement over time in change-of-direction speed suggest change-of-direction ability is related to the processes related to growth and maturation [33] and is trainable. However, consideration of the more complex actions of accelerating, decelerating and re-accelerating may suggest that such development is more complicated than other physical qualities (e.g. linear speed) and further research is required to understand the development and trainability of change of direction.

With respect to playing standard, elite players outperformed sub-elite players [26–28] (e.g. agility 5-0-5; under 17, 2.36 ± 0.08 vs. 2.68 ± 0.08 s) [26], national players were faster than regional players (see Table 2) [13], starters were quicker than non-starters (2.31 ± 0.13 vs. 2.43 ± 0.15 s) [25] and future professionals had greater change-of-direction ability than future amateurs (e.g. 2.42 ± 0.12 vs. 2.52 ± 0.16 s) [14, 36]. This suggests advanced change-of-direction speed contributes to a greater playing standard and future career attainment level. This finding may be expected as the greater change-of-direction ability allows players to better position themselves when attacking (e.g. evading a defender) and defending (e.g. defensive line retreat). Interestingly, change-of-direction speed was found to be the most discriminating factor for career attainment in players aged 13–15 years [14] and therefore may be an important aspect for development within young players.

In most research, positional differences for change-of-direction speed have been less apparent than other physical qualities [24, 26, 27, 39], with only props significantly slower than other positions [17]. Such findings may be apparent due to the regular 180° changes of direction required in all positions during the defensive line retreat, and that such movements may be of a greater quantity and intensity in the forwards, who are closer to the play of the ball. Till et al. [17] suggested that greater change-of-direction speed in backs may be due to the greater body mass and body fat in props, which may increase the eccentric braking capabilities to halt momentum in one direction before immediately accelerating in a new direction [49]. This is a potential explanation why props have lower career attainment success in rugby league [36] (i.e. reduced ability to change direction lessens their ability to meet the increasing movement demands associated with progressing levels of match-play).

In summary, change-of-direction speed has less research available to make comparisons and inform the development of this physical quality. However, findings generally suggest change-of-direction speed increases with age and differentiates between playing standard, but shows less apparent differences between playing positions. Therefore, change-of-direction speed seems an important physical quality for academy player development but future research is required to understand the long-term development and trainability of this quality. In addition, ‘agility’-based research, defined as “*the ability to change direction in response to a specific stimulus*” [50] may be more appropriate than change-of-direction speed alone. Research on agility is available in adult rugby league players [47, 51] demonstrating differences across playing levels, but is not available in youth players and should be a consideration for future research.

## Aerobic Capacity

A well-developed aerobic capacity is important for rugby league performance given the distances covered at low speeds and the imperative need to recover quickly following repeated high-intensity efforts [5]. Aerobic capacity has been assessed via the multistage fitness test [27] or Yo-Yo Intermittent Recovery Test Level 1 [21], and is presented as estimated maximum oxygen uptake ($\dot{V}{\text{O}}_{2\hbox{max} }$) in Table 2. Aerobic capacity has been shown to differentiate between age categories within some studies [17, 20, 23, 24, 26] but not others [19, 21, 25]. Differences between playing level [13, 26–28, 52] are consistent with greater aerobic capacity, contributing to a higher playing standard. For playing position, backs have been shown to have a greater aerobic capacity in some studies [17, 24, 26, 39] but not others [21].

Current research is contradictory as to whether aerobic capacity differentiates between age categories [17, 19–21, 23, 24, 26, 53]. This finding may occur due to methodological considerations such as the testing protocols used or the timing of testing, with most studies using data at the start of the preseason. Longitudinal studies have shown improvements in aerobic capacity with training [44], seasonally [35, 40], annually, and long-term (i.e. 4 years) [34]. This suggests the non-significant differences found in some cross-sectional studies [19, 21, 25] may not represent the development of aerobic capacity with age. However, estimated $\dot{V}{\text{O}}_{2\hbox{max} }$ changes have been shown to be greater in younger (i.e. under 15s) compared with older players (i.e. under 18s) following 10- [19] and 14-week [44] training periods, but not when assessed across a season [35] for under 18s and 20s versus under 14s and 16s. Such findings suggest that aerobic capacity development may be a combination of training-related changes, alongside growth and maturational development, which is impacted upon by training history and current training status [34, 47].

Aerobic capacity has differentiated between elite and sub-elite players [26, 27, 52] (e.g. multistage fitness test level; under 15, 10.6 ± 0.5 vs. 8.0 ± 0.6) [26], contributed to whether a player started a game [25], and discriminated between regional-and national-level players (see Table 2) [13]. Furthermore, advanced aerobic capacity contributed to future career attainment in players in the under 13–15 (amateur 47.6 ± 5.6 vs. professional 49.8 ± 4.6 ml kg^−1^ min^−1^) [14, 36] and under 17–18 age categories (Yo-Yo distance under 17, 1512 ± 299 vs. 1252 ± 262 m) [38]. This suggests that advanced aerobic capacity is an important physical quality for increased playing standard and long-term career attainment success. Advanced aerobic capacity has been shown to contribute to greater high-speed distance covered during a game [54], maintenance of playing intensity across a tournament [55], and quicker recovery following match-play [54], potentially due to reduced metabolic disturbances following intense intermittent exercise [56]. Therefore, advanced aerobic capacity would allow players to maintain playing intensity while increasing training volume and intensity to allow improved player development.

Differences in aerobic capacity between backs and forwards are inconsistent [17, 21, 24, 26, 39]. Like linear speed, forwards may have reduced aerobic capacity compared with backs due to their greater body mass or sum of skinfold measurements (body composition), which may impact on the ability to perform aerobic capacity tests. At least in rugby union, using body mass as a covariate in comparisons across age categories has standardised the difference in aerobic capacity according to body mass [57]. Again, this may be relevant to the match demands of different playing positions, with forwards regularly having a reduced playing time compared with backs [11].

In summary, aerobic capacity increases with age and differentiates between playing position, playing standard and future career attainment. This is an important consideration for the youth rugby league player aged 13–20 years due to its influence on match performance and recovery post-match. Future studies should aim to control for mass in their comparisons of aerobic capacity, and identify optimal methods to maximise the improvement of this quality alongside other important physical characteristics in training periods and annual plans.

## Muscular Strength

Muscular strength is another key attribute for rugby league due to the contact and collision element of the sport [5], alongside its relationship with those for other physical qualities [58]. Strength assessments are less common than other physical qualities, especially in players aged below 16 years. Table 3 shows the strength characteristics of players aged 16–20 years.

**Table 3:** Strength qualities of elite youth rugby league players categorised by age and playing position

Age category (years)	Position	1RM bench press (kg)	Relative bench press (kg.kg^−1^)	1RM squat (kg)	Relative squat (kg kg^−1^)	1RM prone row (kg)	Relative prone row (kg kg^−1^)
Under 16	All [59]	85.0 ± 10.4					
	All [34]	76.5 ± 15.9	0.98 ± 0.16	109.0 ± 23.4	1.40 ± 0.26	73.8 ± 12.2	0.95 ± 0.14
	Backs [21]	70.9 ± 15.0	0.99 ± 0.18	94.9 ± 25.7	1.33 ± 0.32	68.9 ± 11.6	0.96 ± 0.12
	Forwards [21]	76.8 ± 10.9	0.97 ± 0.12	105.2 ± 17.3	1.32 ± 0.19	72.6 ± 8.5	0.92 ± 0.12
Under 17	All [34]	92.7 ± 10.7	1.10 ± 0.13	124.8 ± 16.5	1.48 ± 0.21	86.3 ± 11.9	1.02 ± 0.10
	Backs [21]	89.3 ± 12.6	1.18 ± 0.13	118.1 ± 18.8	1.56 ± 0.22	79.4 ± 10.5	1.05 ± 0.11
	Forwards [21]	96.0 ± 13.6	1.12 ± 0.14	124.9 ± 18.8	1.46 ± 0.22	86.3 ± 9.3	1.01 ± 0.10
Under 18	All [59]	98.2 ± 13.5					
	All [34]	106.6 ± 11.4	1.24 ± 0.14	137.2 ± 15.7	1.60 ± 0.20	94.5 ± 10.8	1.09 ± 0.10
	Backs [21]	98.5 ± 13.8	1.24 ± 0.13	129.6 ± 16.8	1.65 ± 0.18	86.8 ± 10.4	1.09 ± 0.10
	Forwards [21]	107.5 ± 15.3	1.19 ± 0.16	136.9 ± 14.2	1.51 ± 0.17	94.3 ± 8.6	1.04 ± 0.10
Under 19	All [34]	114.6 ± 17.1	1.31 ± 0.19	144.9 ± 15.7	1.66 ± 0.17	100.8 ± 12.4	1.15 ± 0.11
	Backs [21]	110.0 ± 16.3	1.34 ± 0.13	132.1 ± 20.2	1.61 ± 0.18	93.0 ± 12.4	1.13 ± 0.10
	Forwards [21]	115.6 ± 16.3	1.23 ± 0.17	143.7 ± 17.9	1.55 ± 0.23	101.2 ± 11.4	1.08 ± 0.12
Under 20	Backs [43] Forwards [43]	101.7 ± 9.1 110.0 ± 15.8		132.7 ± 9.4 140.0 ± 26.2	1.61 ± 0.13 1.56 ± 0.20		
	Backs [21] Forwards [21]	112.8 ± 15.8 115.5 ± 15.3	1.32 ± 0.15 1.25 ± 0.18	136.9 ± 21.0 151.2 ± 21.6	1.59 ± 0.21 1.65 ± 0.29	97.0 ± 11.0 102.5 ± 11.2	1.13 ± 0.07 1.11 ± 0.11

Data are expressed as mean ± standard deviation

*1RM* one repetition maximum

Both absolute and relative strength have been shown to differentiate between age categories [21, 59, 60]. Longitudinal tracking of strength across a season [34], annually, and long-term (i.e. 4 years) [34] showed improvements with age in academy players. When analysed on an annual basis in terms of age categories (e.g. under 16s–17s, under 18s–19s), Till and colleagues [34] demonstrated greater gains in strength were apparent in the younger (i.e. under 16s–17s) age groups, which may be a result of players being nearer maturation in addition to their first exposures to resistance-based training. Therefore, current available data suggest that strength will increase with age, especially in academy programmes where resistance training is regularly performed.

Limited research is available comparing strength performance according to playing standard, but the available data suggest strength is advantageous to performance level [38, 60]. For example, Till et al. [38] demonstrated that future professional players aged between under 17s and 19s had increased one repetition maximum (1-RM) squat (e.g. under 17, 134.3 ± 12.8 vs. 117.3 ± 20.1 kg), bench press (e.g. under 18, 115.4 ± 15.4 vs. 106.8 ± 14.2 kg) and prone row (e.g. under 19s, 107.4 ± 10.8 vs. 99.0 ± 11.6 kg) when compared with academy players. These strength advantages may result in greater speed and power performance, which have been related to tackling and ball-carrying ability [28, 45], while increased lower body strength has been related to greater running, collision, repeat effort and internal loads in sub-elite match-play and reduced neuromuscular fatigue [54]. Therefore, increased strength leads to greater capacity to undertake match activities and recover from training and match-play, allowing greater training and playing opportunities for long-term player development. As such, advanced strength is beneficial for performance and player development, and may be an essential quality to develop within academy-aged players.

Regarding playing position, forwards have been shown to have increased absolute strength compared with backs, but no differences in relative strength [21, 43]. Absolute strength differences may be apparent due to the positional demands of backs and forwards in that forwards undertake more contact- and collision-based activities during match-play, requiring forwards to have greater absolute strength. As backs predominantly have greater countermovement jump and sprint-speed performance than forwards, it could be expected that a greater relative strength would be apparent due to the moderate relationships between relative strength and sprint performance [43]. However, further data may be required to explore the strength characteristics of youth rugby players.

In summary, strength improves with age, especially when resistance training interventions are implemented, and differentiates between playing level and position. Strength may be an essential physical quality for enhanced playing standard and future career attainment. Although recent recommendations for youth athletes [61, 62] have emphasised the importance of strength development in youth, limited data are available for youth rugby league players. Future research should aim to evaluate strength within such populations, with the possible use of the isometric mid-thigh pull that allows implementation of strength assessments with a reduction in technical competency (i.e. the squat) [63]. Furthermore, the understanding of appropriate strength training interventions should be prioritised alongside the links between strength and injury.

## Muscular Power

Muscular power is a key attribute for rugby league due to the contact and collision element of the sport [5, 64]. Lower body power has been assessed, although indirectly, consistently in the literature via the countermovement jump, with data presented in Table 2. Upper body power assessments are less common, with the medicine ball chest throw having been used to assess upper body power in 13- to 15-year-olds but no studies having assessed upper body power in players aged >16 years. Both upper and lower body muscular power have been shown to differentiate between age categories [17, 19–21, 24, 26, 60]. Studies have demonstrated greater lower body power with increased playing standard [13, 26–28], although this is not consistent for upper body power [13]. Muscular power differences between playing positions are inconsistent with some studies showing backs to be greater than forwards for lower body power [17, 20, 21, 26], but others showing no differences [24, 39, 43]. For upper body power, forwards outperform backs [17, 20].

Current research shows that both upper and lower body muscular power (countermovement jump height) differentiates and increases between age categories [17, 19–21, 24–26, 57]. The only study where jump height did not increase was in players aged under 15–17 years [23], despite increases in peak power between these time points. Studies assessing lower body power longitudinally demonstrated improvements in performance across 10 [19] and 14 weeks [44] of training, across a season [35, 40], annually, and long-term (i.e. 4 years) [34]. Greater improvement in lower body power was apparent when younger players (e.g. under 15s) were compared with older players (e.g. under 18s) [19, 21, 44]. These changes in power are associated with the normal adaptations related to growth and maturation alongside the implementation of resistance training that is common during this period, especially in older youth players (i.e. post 16 years) [21].

Lower body power has demonstrated differences between elite and sub-elite players [26–28] (e.g. under 16, 53.1 ± 3.6 vs. 40.3 ± 3.3 cm) [26], national and regional players (see Table 2) [13] and between future levels of career attainment (e.g. amateur 39.2 ± 4.9 vs. professional 41.2 ± 7.2 cm) [14, 36]. Such differences between playing standards may be apparent due to the relationship between vertical jump performance and tackling ability [28] and successful ball carries [45] in players aged 15–17 years. However, upper-body-power studies have failed to demonstrate differences between national and regional players (see Table 2) [13], with future professionals actually underperforming compared with future amateurs at 13–15 years (e.g. under 14, professional 5.0 ± 0.7 vs. academy 5.8 ± 0.8 m) [36]. This finding may suggest that lower body power compared with upper body power may be a more important physical quality at younger ages (i.e. under 16 years). However, limited studies are available evaluating upper body power post 16 years [57].

Regarding playing position, comparisons between backs and forwards are inconsistent with some studies showing backs are greater than forwards [17, 20, 21, 26] for lower body power, whereas others show no difference [24, 39, 43]. For upper body power, forwards outperform backs between 13 and 15 years of age [17, 20]. Such findings may again be due to the increased mass and body composition of forwards, which affects the ability to exert force explosively. Therefore, it may be more appropriate to monitor power output than jump height per se when monitoring power development, but few studies have utilised such an approach [59, 65].

In summary, lower and upper body power seem to increase with age, and lower body power seems to be an important physical quality to differentiate between playing standard and future career attainment; therefore, appropriate monitoring and development strategies should be implemented to monitor this characteristic. Upper body power seems less important due to its strong relationship with maturation [17, 22], with further data needed in relation to this physical quality post 16 years and utilising better methodologies than a medicine ball throw.

## Maturation

Although age has been considered throughout this review, another important factor in player development is maturation status, which has been considered in some research in youth rugby league players [13, 22, 23]. Maturation is the timing and tempo of progress towards the mature adult state, which can vary considerably between individuals during adolescence [33, 66]. Based on these individual variations, youths can be viewed as biologically ahead (early maturer), on time (average maturer) or behind (late maturer) their peers [66]. Maturity status can be estimated by calculating age at PHV [67]. Age at PHV was 13.61 ± 0.58 years in UK youth rugby league players, indicating earlier maturation compared with European boys (e.g. 14.2 years) [33]. Differences have been identified between playing positions with forwards (props 13.29 ± 0.43, back row 13.41 ± 0.49 years) generally maturing earlier than backs (outside backs 13.66 ± 0.54, pivots 14.00 ± 0.96 years), which may suggest that players are assigned playing positions based on their maturation status.

Some studies [13, 68, 69] have demonstrated the relationships between maturation and physical qualities, with the maturity offset group (players grouped according to years from PHV [YPHV] rather than chronological age) impacting on all anthropometric and physical variables (e.g. 20 m, −2.5 YPHV 3.46 ± 0.08, −0.5 YPHV 3.28 ± 0.15, 1.5 YPHV 3.17 ± 0.13 s) except multistage fitness test performance. These findings are explained by the increased testosterone [33], muscle volume and size [70], and qualitative changes in muscle (e.g. contractile properties) [71] associated with advanced maturation. Furthermore, longitudinal research [22] has demonstrated greater improvements in anthropometric and physical qualities in later-maturing players compared with earlier-maturing players over a 2 year period between 13 and 15 years of age (e.g. change in height, late maturers 10.3, early maturers 5.0 cm). This suggests that later-maturing players have greater potential for growth and performance development during adolescence and, therefore, maturation should be considered when monitoring and evaluating physical performance, especially within younger players (i.e. below 16 years of age).

## Conclusions, Limitations and Further Research

This review provides a first attempt to present current evidence on the anthropometric and physical qualities of elite youth male rugby league players, considering age, playing standard and positions. A plethora of research exists for youth rugby league players (i.e. anthropometry, 12 studies; linear speed, change-of-direction speed, power and aerobic capacity, 11 studies; strength, 4 studies) matching the inclusion criteria (i.e. elite standard by playing position) and is presented in Tables 1, 2, 3. The under 15s (i.e. national programme) [13, 17] have enhanced physical qualities compared with players in the under 16 age category (from one professional club academy), potentially questioning the appropriateness of the current literature in providing normative data for the anthropometric and physical qualities of youth rugby league players aged 16–20 years. It is therefore recommended that a national standardised testing battery in the UK and Australasia be developed and implemented to provide practitioners with normative data, especially post 16 years, to support the identification and physical development of youth players.

Although the lack of a national dataset post 16 years is a limitation of the current available research, there is a large range of studies utilising multiple research methodologies (i.e. cross-sectional, longitudinal, retrospective research designs) to describe the influence of age, playing standard and position on anthropometric and physical qualities. Age and maturation have a significant impact on the development of such qualities from 13 to 20 years of age, with only body composition (across 13–20 years of age) and linear speed (16–20 years of age) failing to improve with age, most probably due to the very large interplayer variability in these measures [34]. This is an important consideration for practitioners, and it is recommended that such qualities are closely monitored and evaluated throughout this youth period. In addition, practitioners should understand and monitor the use of combined anthropometric and physical qualities (e.g. momentum).

Anthropometric and physical qualities differentiated across playing standards and career attainment levels emphasise the importance of physical qualities to rugby league success. This highlights the importance of anthropometric and physical qualities for informing both talent identification and development purposes. However, it is recommended that both practice and future research include maturity in assessment protocols, especially in players under 16 years of age. Future work should also aim to develop innovative statistics to help understand the contribution of characteristics to success, alongside how such physical qualities influence the specificity and sensitivity of success [72]. Furthermore, longitudinal assessments and monitoring should be implemented as they provide data in relation to current performance and developmental change that are of importance for playing progression and informing decisions. Where possible, this could be implemented on an individual basis [68, 73].

The implementation of strength and conditioning practices is essential for the development of youth rugby league players. Current research suggests a range of physical qualities influence career attainment at 13–15 years of age. Therefore, practitioners should implement physical training programmes including speed, change of direction (‘agility’), aerobic capacity, and muscular strength and power to provide a broad base of physical development [74]. However, comparisons between youth and senior players show strength and size [30] are the main discriminating factors within 16- to 19-year-old academy players, while body composition and strength were the primary physical qualities that contributed to attaining professional status [38]. This suggests that upon commencement in an academy-based programme, where strength and conditioning support is available, resistance-based training interventions should be a major focus to develop strength and size, alongside speed, change of direction and aerobic capacity qualities. Recent research shows that strong relationships exist between strength and other physical qualities (i.e. speed, agility, power, aerobic capacity) [75], suggesting improved strength performance would result in improvements in other qualities. The implementation of multi-joint exercises involving squats, Olympic lifts and plyometrics may be recommended [76].

The importance of physical qualities for the development of rugby league players is clear. However, future research is still required to optimise talent identification and development programmes. Current research [17, 20, 21, 23–27] is limited as it has not explored the role that the dose–response relationship of training exposure plays in the physical development of rugby league players, with few intervention studies currently available. Future research should include intervention-based studies or should quantify the training load of athletes to understand the most appropriate strategies for enhancing physical qualities. Next, studies need to understand the relationship between physical qualities and match performance while providing greater consideration of the holistic development of the youth rugby league player, including technical skill, tactical knowledge, psychological characteristics, and injury occurrence and reduction [4].
